# Establishment and validation of multiclassification prediction models for pulmonary nodules based on machine learning

**DOI:** 10.1111/crj.13769

**Published:** 2024-05-12

**Authors:** Qiao Liu, Xue Lv, Daiquan Zhou, Na Yu, Yuqin Hong, Yan Zeng

**Affiliations:** ^1^ Department of Radiology The Third Affiliated Hospital of Chongqing Medical University Chongqing China

**Keywords:** machine learning (ML), prediction model, probability of malignancy, pulmonary nodules (PNs)

## Abstract

**Background:**

Lung cancer is the leading cause of cancer‐related death worldwide. This study aimed to establish novel multiclassification prediction models based on machine learning (ML) to predict the probability of malignancy in pulmonary nodules (PNs) and to compare with three published models.

**Methods:**

Nine hundred fourteen patients with PNs were collected from four medical institutions (A, B, C and D), which were organized into tables containing clinical features, radiologic features and laboratory test features. Patients were divided into benign lesion (BL), precursor lesion (PL) and malignant lesion (ML) groups according to pathological diagnosis. Approximately 80% of patients in A (total/male: 632/269, age: 57.73 ± 11.06) were randomly selected as a training set; the remaining 20% were used as an internal test set; and the patients in B (total/male: 94/53, age: 60.04 ± 11.22), C (total/male: 94/47, age: 59.30 ± 9.86) and D (total/male: 94/61, age: 62.0 ± 11.09) were used as an external validation set. Logical regression (LR), decision tree (DT), random forest (RF) and support vector machine (SVM) were used to establish prediction models. Finally, the Mayo model, Peking University People's Hospital (PKUPH) model and Brock model were externally validated in our patients.

**Results:**

The AUC values of RF model for MLs, PLs and BLs were 0.80 (95% CI: 0.73–0.88), 0.90 (95% CI: 0.82–0.99) and 0.75 (95% CI: 0.67–0.88), respectively. The weighted average AUC value of the RF model for the external validation set was 0.71 (95% CI: 0.67–0.73), and its AUC values for MLs, PLs and BLs were 0.71 (95% CI: 0.68–0.79), 0.98 (95% CI: 0.88–1.07) and 0.68 (95% CI: 0.61–0.74), respectively. The AUC values of the Mayo model, PKUPH model and Brock model were 0.68 (95% CI: 0.62–0.74), 0.64 (95% CI: 0.58–0.70) and 0.57 (95% CI: 0.49–0.65), respectively.

**Conclusions:**

The RF model performed best, and its predictive performance was better than that of the three published models, which may provide a new noninvasive method for the risk assessment of PNs.

## Graphical abstract

The RF model based on machine learning (ML) performed best, and its predictive performance was better than that of the three published models include Mayo model, Peking University People's Hospital (PKUPH) model and Brock model, which may provide a new noninvasive method for the risk assessment of PNs.

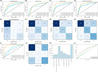

## 1 INTRODUCTION

Lung cancer is the leading cause of cancer‐related death worldwide. According to GLOBOCAN data, lung cancer caused approximately 1.8 million deaths in 2020, which constituted 18% of cancer‐related deaths.^1^ In 2015, there were approximately 0.73 million new cases of lung cancer and approximately 0.61 million deaths from lung cancer in China.^2^ Due to population aging, environmental pollution and smoking, the incidence and mortality of lung cancer are expected to further increase.^3^, ^4^ Early detection, diagnosis and treatment are key to reducing mortality from lung cancer.^5^

In recent years, thanks to the wide use of low‐dose computer tomography (LDCT) screening, the detection rate of PNs has increased to 51%.^6^ The National Lung Cancer Screening Trial (NLST) reported that LDCT screening reduced mortality from lung cancer by approximately 20% in a high‐risk population compared with chest radiography.^7^ However, more than 90% of these nodules are benign, which causes a high number of false‐positive results.^6^ This can lead to anxiety and additional imaging tests and invasive diagnostic procedures.^6^, ^7^, ^8^ Therefore, it is very important to find a method to accurately diagnose PNs.

In routine clinical practice, the preoperative diagnosis of PNs mainly depends on clinicians' experience; thus, the diagnosis results are greatly affected by subjective factors. In addition, biopsy is the gold standard for the diagnosis of PNs, but this method is invasive. In response to these problems, many researchers have established mathematical models to diagnose PNs in an objective and noninvasive way.

The Mayo model,^9^ Brock model,^10^ and Peking University People's Hospital (PKUPH) model^11^ are the most commonly used and have been extensively validated. However, these models have some limitations. In the Mayo model, 12% of patients had unclear pathological diagnoses. The Brock model was only recommended for current and former smokers between 50 and 75 years of age without a history of lung cancer. The predictive features of these models were only clinical and imaging features, without laboratory test features. In addition, these models were based on only the logistic regression (LR) algorithm. In recent years, other machine learning (ML) algorithms, such as random forest (RF) and support vector machine (SVM), have been widely used in medical research and perform outstanding in disease diagnosis, risk assessment, prediction outcomes and other factors.^12^, ^13^, ^14^, ^15^ They are expected to provide novel methods for risk assessment of PNs. Therefore, this study aimed to establish multiclassification ML models for the noninvasive prediction of PNs based on clinical features, imaging features and laboratory test data. We also evaluated the predictive performance of the Mayo model, Brock model and PKUPH model in our patients. We present the following article in accordance with the TRIPOD reporting checklist.

## 2 METHODS

### 2.1 Patients

This was a multicentre retrospective study. We recruited 914 PN patients from four medical institutions (A, B, C and D) in Chongqing between January 2013 and October 2021. Patients from hospital A (total/male: 632/269, age: 57.73 ± 11.06) were used as the development cohort and were randomly divided into a training set and an internal test set at a ratio of 8:2. Patients from hospitals B (total/male: 94/53, age: 60.04 ± 11.22), C (total/male: 94/47, age: 59.30 ± 9.86) and D (total/male: 94/61, age: 62.0 ± 11.09) were used as an external validation set. The training set was used for feature selection and model training. The internal test set and external validation set were used to validate the predictive performance of the models.

The inclusion criteria were as follows: (1) the maximal diameter of the nodule was less than 30 mm; (2) the diagnosis of the nodule was pathologically confirmed through operation or biopsy; and (3) the nodule was found to have been radiographically stable for at least 2 years. The exclusion criteria were as follows: (1) prior chemotherapy, radiotherapy or surgical treatment; (2) a history of thoracic cancer or extrathoracic malignant neoplasm within 5 years; (3) nodules with atelectasis or the presence of pleural effusion; and (4) a metastatic tumour. According to pathological diagnosis, all the patients were divided into the benign lesion (BL) group, the precursor lesion (PL) group and the malignant lesion (ML) group.

### 2.2 Data collection and cleaning

Based on electronic medical records, we collected 79 features relating to clinical features, radiologic features and laboratory test features. In the development cohort, features with a missing rate greater than 20% were removed, and features with a missing rate less than 20% were populated by a mean value or a mode. Finally, eight features were deleted because of missing data, and 71 features were included for analysis.

### 2.3 Statistical analysis

Data processing involved the following steps. In the first step, univariate analysis was performed to compare the differences in each feature between groups. Continuous variables with a normal distribution are expressed as the means ± standard deviation (SD); otherwise, they are expressed as the medians and interquartile range. Categorical variables were reported as the counts with percentages. According to the distribution of normality and homogeneity of variance, group comparisons of continuous variables were analysed with the analysis of variance (ANOVA) test or Kruskal–Wallis test. Group comparisons of categorical variables were analysed with the chi‐square test or Fisher's exact test; *p* < 0.05 was considered statistically significant. In the second step, the statistically significant features in the univariate analysis were further screened by recursive feature elimination (RFE). In the third step, the selected features were incorporated into the ML models to establish the prediction models, including LR, decision tree (DT), RF and SVM. The accuracy, precision, recall, F1 score, receiver operating characteristic (ROC) curve and area under the curve (AUC) of the models were calculated. Because the predicted types of this study had three categories, and the categories were imbalanced, we compared the predictive performance of the models by weighted average AUC. Finally, the predictive performance of the Mayo model, PKUPH model and Brock model was externally validated in our patients. The descriptions of these three models are shown in Table S1.

### 2.4 Ethical statement

The study was conducted in accordance with the Declaration of Helsinki (as revised in 2013). The study was approved by the Ethics Committee of the Third Affiliated Hospital of Chongqing Medical University (2022‐41), and individual consent for this retrospective analysis was waived.

## 3 RESULTS

### 3.1 Baseline characteristics

**FIGURE 1:**
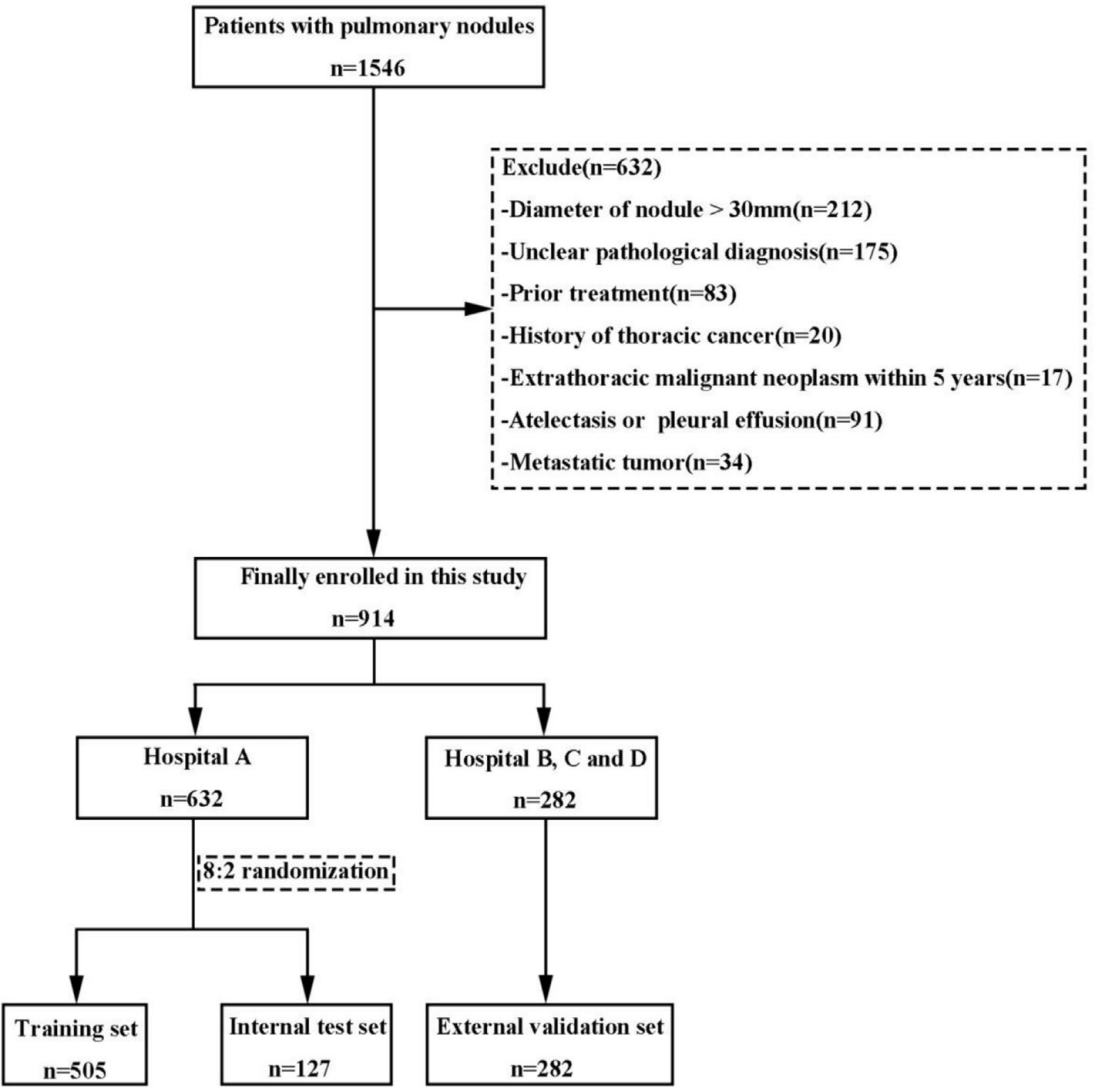
Flow diagram of patient selection.

Overall, 914 patients with PNs were enrolled in this study, including 430 (47.05%) males and 484 (52.95%) females, aged 58.57 ± 11.04 years. The training set, internal test set and external validation set contained 505 cases, 127 cases and 282 cases, respectively (Figure 1). There were 250 (27.35%) BLs, 103 (11.27%) PLs and 561 (61.38%) MLs. The pathological diagnoses of PNs are shown in Table S2.

### 3.2 Predictive feature selection

Predictive feature selection was based on the training set. Table 1 provides the results of the univariate analysis for all 71 features considered as potential predictors in our study. Thirty‐one candidate predictive features with *p* < 0.05 were used as the input data in the REF. Finally, eight features were selected as the predictive features of this study, including age, maximum nodule diameter, nodule type, carcinoembryonic antigen (CEA), cytokeratin 19 fragment (CYFRA21‐1), platelet large cell ratio (P‐LCR), mean corpuscular haemoglobin concentration (MCHC) and percentage of monocytes (MONO%). The features of patients in the internal test set and external validation are described in Table S3.

**TABLE 1:** The results of univariate analysis for the training set.

Features	Training set (*n* = 505)			*p* value
	ML group (*n* = 316)	PL group (*n* = 74)	BL group (*n* = 115)	
**Clinical features**				
Gender				<0.001^c^
Female	184 (58.2)	57 (77.0)	54 (47.0)	
Male	132 (41.8)	17 (23.0)	61 (53.0)	
Age (years)	62.00 (53.75–68.00)	52.50 (45.00–58.00)	53.00 (46.50–62.00)	<0.001^b^
Smoking history				<0.001^c^
No	207 (65.5)	68 (91.9)	72 (62.6)	
Yes	109 (34.5)	6 (8.1)	43 (37.4)	
Number of years of smoking (years)	0.00 (0.00–30.00)	0.00 (0.00–0.00)	0.00 (0.00–20.00)	<0.001^b^
Number of cigarettes per day smoked (cigarettes)	0.00 (0.00–20.00)	0.00 (0.00–0.00)	0.00 (0.00–15.00)	<0.001^b^
Family history of cancer				0.84^c^
No	288 (91.1)	68 (91.9)	103 (89.6)	
Yes	28 (8.9)	6 (8.1)	12 (10.4)	
History of hypertension				<0.001^c^
No	219 (69.3)	64 (86.5)	94 (81.7)	
Yes	97 (30.7)	10 (13.5)	21 (18.3)	
History of diabetes				0.88^c^
No	283 (89.6)	66 (89.2)	101 (87.8)	
Yes	33 (10.4)	8 (10.8)	14 (12.2)	
History of coronary heart disease				0.01^c^
No	288 (91.1)	74 (100.0)	110 (95.7)	
Yes	28 (8.9)	0 (0.0)	5 (4.3)	
History of viral hepatitis				0.51^d^
No	307 (97.2)	73 (98.6)	110 (95.7)	
Yes	9 (2.8)	1 (1.4)	5 (4.3)	
History of hyperlipidaemia				0.91^c^
No	295 (93.4)	70 (94.6)	107 (93.0)	
Yes	21 (6.6)	4 (5.4)	8 (7.0)	
History of pulmonary tuberculosis				0.38^d^
No	309 (97.8)	71 (95.9)	110 (95.7)	
Yes	7 (2.2)	3 (4.1)	5 (4.3)	
History of chronic obstructive pulmonary disease				0.30^d^
No	306 (96.8)	74 (100.0)	113 (98.3)	
Yes	10 (3.2)	0 (0.0)	2 (1.7)	
History of cancer				0.09^d^
No	314 (99.4)	72 (97.3)	112 (97.4)	
Yes	2 (0.6)	2 (2.7)	3 (2.6)	
Systolic blood pressure (mmHg)	132.08 ± 20.54	125.86 ± 19.03	130.49 ± 18.15	0.05^a^
Diastolic blood pressure (mmHg)	77.45 ± 11.45	76.09 ± 10.13	77.86 ± 10.15	0.54^a^
Pulse (beats/minute)	75 (68.0–82.3)	74 (67.0–82.0)	76 (67.0–85.0)	0.48^b^
Temperature (°C)	36.50 (36.40–36.60)	36.50 (36.40–36.60)	36.50 (36.40–36.65)	0.57^b^
Respiratory rate (breaths/minute)	20 (19.0–20.0)	20 (18.3–20.0)	20 (18.0–20.0)	<0.001^b^
**Radiologic features**				
Nodule location				0.57^c^
Upper right	96 (30.4)	22 (29.7)	28 (24.3)	
Lower right	57 (18.0)	7 (9.5)	26 (22.6)	
Middle right	22 (7.0)	5 (6.8)	8 (7.0)	
Upper left	85 (26.9)	25 (33.8)	32 (27.8)	
Lower left	56 (17.7)	15 (20.3)	21 (18.3)	
Nodule type				<0.001^c^
Solid	137 (43.4)	7 (9.5)	93 (80.9)	
Part‐solid	111 (35.1)	25 (33.8)	14 (12.2)	
Pure ground‐glass	68 (21.5)	42 (56.8)	8 (7.0)	
Maximal diameter of nodule (mm)	16.00 (11.95–21.25)	9.25 (7.00–11.00)	12.00 (8.20–19.00)	<0.001^b^
Clear boundary				0.004^c^
No	280 (88.6)	63 (85.1)	87 (75.7)	
Yes	36 (11.4)	11 (14.9)	28 (24.3)	
Regular edges				<0.001^c^
No	306 (96.8)	70 (94.6)	99 (86.1)	
Yes	10 (3.2)	4 (5.4)	16 (13.9)	
Shaggy boundary				0.32^c^
No	299 (94.6)	73 (98.6)	110 (95.7)	
Yes	17 (5.4)	1 (1.4)	5 (4.3)	
Lobulation				0.001^c^
No	237 (75.0)	69 (93.2)	98 (85.2)	
Yes	79 (25.0)	5 (6.8)	17 (14.8)	
Spiculation				<0.001^c^
No	222 (70.3)	72 (97.3)	88 (76.5)	
Yes	94 (29.7)	2 (2.7)	27 (23.5)	
Pleural indentation				<0.001^c^
No	241 (76.3)	73 (98.)	96 (83.5)	
Yes	75 (23.7)	1 (1.4)	19 (16.5)	
Adjacent pleural thickening				0.26^d^
No	305 (96.5)	74 (100.0)	111 (96.5)	
Yes	11 (3.5)	0 (0.0)	4 (3.5)	
Vacuole sign				<0.001^c^
No	272 (86.1)	73 (98.6)	112 (97.4)	
Yes	44 (13.9)	1 (1.4)	3 (2.6)	
Cavity				0.05^d^
No	311 (98.4)	74 (100.0)	109 (94.8)	
Yes	5 (1.6)	0 (0.0)	6 (5.2)	
Calcification				<0.001^d^
No	312 (98.7)	74 (100.0)	105 (91.3)	
Yes	4 (1.3)	0 (0.0)	10 (8.7)	
Enlargement of hilar or mediastinal lymph nodes				0.004^c^
No	288 (91.1)	73 (98.6)	97 (84.3)	
Yes	28 (8.9)	1 (1.4)	18 (15.7)	
Number of nodule				0.30^c^
Solitary	91 (28.8)	24 (32.4)	42 (36.5)	
Multiple	225 (71.2)	50 (67.6)	73 (63.5)	
**Laboratory test**				
CEA (ng/mL)	2.45 (1.72–3.76)	1.46 (1.00–2.33)	2.33 (1.31–3.76)	<0.001^b^
Pro‐GRP (pg/mL)	57.22 (43.91–63.70)	51.68 (39.88–58.34)	55.65 (43.00–59.67)	0.12^b^
NSE (ng/mL)	16.71 (12.46–18.64)	17.07 (12.11–19.17)	17.24 (12.21–18.44)	0.95^b^
CYFRA21‐1 (ng/mL)	2.31 (1.68–2.78)	2.02 (1.53–2.31)	2.04 (1.49–2.31)	0.003^b^
P‐LCR (%)	31.70 ± 10.05	31.62 ± 9.71	29.02 ± 7.95	0.03^b^
P‐LCC (10^9^/L)	59.17 ± 16.84	60.66 ± 16.64	60.26 ± 16.89	0.71^a^
MONO% (%)	5.50 (4.60–6.60)	5.05 (4.50–6.18)	5.70 (4.85–6.60)	0.03^b^
RDW‐CV (%)	12.90 (12.40–13.50)	12.70 (12.33–13.20)	12.80 (12.40–13.10)	0.05^b^
RDW‐SD (fL)	44.40 (42.60–46.40)	43.90 (42.02–45.68)	43.70 (41.85–45.50)	0.03^b^
HCT (%)	41.89 ± 4.47	40.70 ± 3.87	42.07 ± 4.30	0.07^a^
LYM% (%)	27.28 ± 8.08	29.43 ± 8.64	27.88 ± 8.47	0.13^a^
MCV (fL)	93.20 (90.57–95.90)	93.60 (90.75–95.88)	92.30 (90.25–95.85)	0.57^b^
MCH (pg)	30.75 (29.8–31.60)	30.75 (29.8–31.58)	30.7 (29.65–31.85)	0.95^b^
MCHC (g/L)	328.92 ± 10.14	328.04 ± 11.43	331.65 ± 9.63	0.02^a^
MPV (fL)	10.70 (9.70–11.60)	10.70 (9.80–11.28)	10.20 (9.70–11.10)	0.04^b^
BAS% (%)	0.40 (0.30–0.60)	0.40 (0.30–0.50)	0.40 (0.30–0.50)	0.85^b^
EOS% (%)	1.70 (0.90–2.90)	1.45 (0.83–2.50)	1.90 (1.20–3.60)	0.10^b^
PDW (%)	16.30 (16.10–16.50)	16.20 (16–16.40)	16.28 (16.10–16.50)	0.13^b^
PCT (%)	0.21 ± 0.05	0.21 ± 0.04	0.22 ± 0.05	0.12^a^
NEU% (%)	63.95 ± 9.13	62.56 ± 9.86	63.07 ± 9.67	0.43^a^
WBC (10^9/L)	5.50 (4.54–6.65)	5.12 (4.39–6.04)	5.52 (4.86–6.66)	0.12^b^
EOS# (10^9/L)	0.09 (0.05–0.16)	0.09 (0.03–0.12)	0.10 (0.06–0.18)	0.11^b^
BAS# (10^9/L)	0.02 (0.02–0.03)	0.02 (0.02–0.03)	0.02 (0.02–0.03)	0.38^b^
NEU# (10^9/L)	3.43 (2.75–4.58)	3.15 (2.63–3.98)	3.50 (2.85–4.45)	0.15^b^
LYM# (10^9/L)	1.47 (1.16–1.77)	1.50 (1.20–1.81)	1.48 (1.25–1.81)	0.55^b^
MONO# (10^9/L)	0.31 (0.24–0.39)	0.27 (0.21–0.34)	0.32 (0.26–0.37)	0.002^b^
PLT (10^9/L)	191.00 (161.75–228.00)	195.00 (155.25–234.75)	209.00 (175.50–248.00)	0.02^b^
HGB (g/L)	137.93 ± 15.41	133.31 ± 13.80	139.61 ± 15.24	0.02^a^
RBC (10^12/L)	4.54 ± 0.51	4.46 ± 0.52	4.57 ± 0.45	0.35^a^
APTT (s)	31.21 ± 3.18	31.69 ± 3.39	31.59 ± 2.63	0.15^b^
TT (s)	14.60 (13.8–15.20)	14.90 (14.30–15.50)	14.60 (13.90–15.20)	0.03^b^
PTA (%)	105.00 (96.00–111.00)	101.50 (97.00–107.00)	102.00 (94.50–109.00)	0.08^b^
PT (s)	11.00 (10.60–11.50)	11.05 (10.90–11.50)	11.20 (10.75–11.65)	0.19^b^
FIB (g/L)	2.89 (2.60–3.26)	2.74 (2.44–2.99)	2.89 (2.56–3.30)	0.005^b^
D‐DimerHS (ng/mL)	88.00 (54.75–133.00)	82.50 (44.25–119.90)	80.00 (51.50–119.90)	0.12^b^
INR	0.97 (0.93–1.01)	0.97 (0.96–1.01)	0.98 (0.94–1.02)	0.18^b^
ALB (g/L)	43.72 ± 4.22	44.52 ± 3.85	43.51 ± 3.87	0.23^a^

*Note*: Data are expressed as mean ± standard deviation or medians and interquartile range or counts with percentages.

Abbreviations: ALB, albumin; APTT, activated partial thromboplastin time; BAS%, percentage of basophils; BAS, basophil count; BL, benign lesion; CEA, carcinoembryonic antigen; CYFRA21‐1, cytokeratin 19 fragment; D‐DimerHS, hypersensitive D‐Dimer; EOS%, percentage of eosinophils; EOS, eosinophil count; FIB, fibrinogen; HCT, haematocrit; HGB, haemoglobin; INR, international normalized ratio; LYM%, percentage of lymphocytes; LYM, lymphocyte count; MCH, mean corpuscular haemoglobin; MCHC, mean corpuscular haemoglobin concentration; MCV, mean corpuscular volume; ML, malignant lesion; MONO%, percentage of monocytes; MONO, monocyte count; MPV, mean platelet volume; NEU%, percentage of neutrophil; NEU, neutrophil count; NSE, neuron‐specific enolase; PCT, plateletcrit; PDW, platelet distribution width; PL, precursor lesion; P‐LCC, platelet large cell count; P‐LCR, platelet large cell ratio; PLT, platelets; ProGRP, pro‐gastrin‐releasing peptide; PT, prothrombin time; PTA, prothrombin activity; RBC, red blood cell; RDW‐CV, red blood cell distribution width coefficient variation; RDW‐SD, red blood cell distribution width standard deviation; TT, thrombin time; WBC, white blood cell.

^a^ Calculated using analysis of variance (ANOVA) test.

^b^ Calculated using Kruskal–Wallis test.

^c^ Calculated using Chi‐square test.

^d^ Calculated using Fisher's exact test.

### 3.3 Validation of the novel prediction models

**FIGURE 2:**
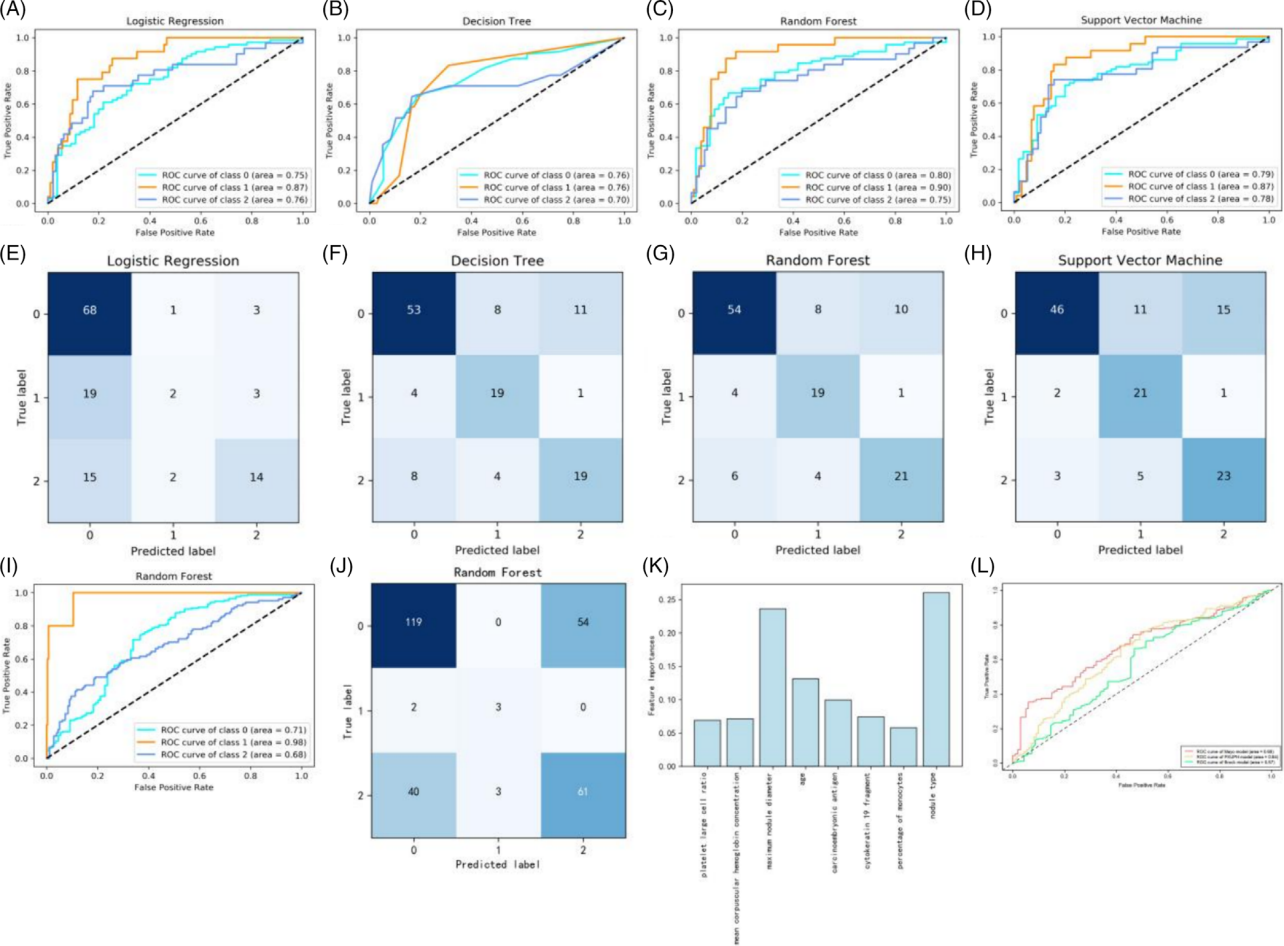
Validation of models (A–H) ROC curve and confusion matrix of the machine learning models for the internal test set. (I, J) ROC curve and confusion matrix of the random forest model for the external validation set. (K) Feature importance derived from random forest model. (L) ROC curve of the Mayo model, PKUPH model and Brock model for pulmonary nodules in our patients. 0, 1 and 2 represent malignant lesions, precursor lesions and benign lesions, respectively. PKUPH, Peking University People's Hospital; ROC curve, receiver operating characteristic curve.

Four ML models were established based on the predictive features. Table 2 and Figure 2A–H show the results of the four ML models for the internal test set. We mainly compared the sizes of the weighted average AUC values. The weighted average AUC values of LR, DT, RF, and SVM were 0.78 (95% CI: 0.75–0.82), 0.75 (95% CI: 0.70–0.86), 0.81 (95% CI: 0.77–0.83) and 0.80 (95% CI: 0.74–0.82), respectively, among which the RF model showed the best predictive performance, and its AUC values of MLs, PLs, and BLs were 0.80 (95% CI: 0.73–0.88), 0.90 (95% CI: 0.82–0.99) and 0.75 (95% CI: 0.67–0.88), respectively. Table 3 and Figure 2I, J show the RF results for the external validation set. Its weighted average AUC value was 0.71 (95% CI: 0.67–0.73), and its AUC values for MLs, PLs, and BLs were 0.71 (95% CI: 0.68–0.79), 0.98 (95% CI: 0.88–1.07) and 0.68 (95% CI: 0.61–0.74), respectively. The results for the external validation set of LR, DT and SVM are shown in Table S4.

**TABLE 2:** Statistics for machine learning models for the internal test set.

Model	Type	Accuracy	Precision	Recall	F1 score	AUC (95% CI)
LR		0.66				
	MLs		0.67	0.94	0.78	0.75 (0.72–0.87)
	PLs		0.40	0.08	0.14	0.87 (0.78–0.97)
	BLs		0.70	0.45	0.55	0.76 (0.65–0.86)
	Weighted average		0.62	0.66	0.60	0.78 (0.75–0.82)
DT		0.72				
	MLs		0.82	0.74	0.77	0.76 (0.70–0.86)
	PLs		0.61	0.79	0.69	0.76 (0.76–0.96)
	BLs		0.61	0.61	0.61	0.70 (0.68–0.89)
	Weighted average		0.73	0.72	0.72	0.75 (0.70–0.86)
RF		0.74				
	MLs		0.84	0.75	0.79	0.80 (0.73–0.88)
	PLs		0.61	0.79	0.69	0.90 (0.82–0.99)
	BLs		0.66	0.68	0.67	0.75 (0.67–0.88)
	Weighted average		0.75	0.74	0.74	0.81 (0.77–0.83)
SVM		0.71				
	MLs		0.90	0.64	0.75	0.79 (0.69–0.85)
	PLs		0.57	0.88	0.69	0.87 (0.79–0.97)
	BLs		0.59	0.74	0.66	0.78 (0.67–0.88)
	Weighted average		0.76	0.71	0.71	0.80 (0.74–0.82)

Abbreviations: 95% CI, 95% confidence interval; AUC, area under the curve; BLs, benign lesions; DT, decision tree; LR, logical regression; MLs, malignant lesions; PLs, precursor lesions; RF, random forest; SVM, support vector machine.

**TABLE 3:** Statistics for machine learning models for the external validation set.

Model	Type	Accuracy	Precision	Recall	F1 score	AUC (95% CI)
RF		0.65				
	MLs		0.74	0.69	0.71	0.71 (0.68–0.79)
	PLs		0.50	0.60	0.55	0.98 (0.88–1.07)
	BLs		0.53	0.59	0.56	0.68 (0.61–0.74)
	Weighted average		0.66	0.65	0.65	0.71 (0.67–0.73)

Abbreviations: 95% CI, 95% confidence interval; AUC, area under the curve; BLs, benign lesions; MLs, malignant lesions; PLs, precursor lesions; RF, random forest.

To enhance model interpretability, we ranked the importance of features. Figure 2K shows the ranking of feature importance for all features in the RF model. Nodule type and maximal diameter were the top two important features in the model, which made a great contribution to the prediction results.

### 3.4 Validation of published models

According to the inclusion and exclusion criteria of each model, 302, 337 and 252 cases met the criteria of the Mayo model, PKUPH model and Brock model, respectively. The AUC values of the Mayo model, PKUPH model and Brock model were 0.68 (95% CI: 0.62–0.74), 0.64 (95% CI: 0.58–0.70) and 0.57 (95% CI: 0.49–0.65), respectively. Other validation results of these models are shown in Table 4 and Figure 2L.

**TABLE 4:** Statistics for published models for our patients.

Model	Accuracy	Precision	Recall	F1 score	AUC (95% CI)
Mayo model	0.55	0.92	0.35	0.51	0.68 (0.62–0.74)
PKUPH model	0.67	0.76	0.75	0.75	0.64 (0.58–0.70)
Brock model	0.63	0.79	0.66	0.72	0.57 (0.49–0.65)

Abbreviations: 95% CI, 95% confidence interval; AUC, area under the curve; PKUPH, Peking University People's Hospital.

## 4 DISCUSSION

In this study, we retrospectively collected 914 patients with PNs from four centers and established four multiclassification ML models to predict the probability of malignancy in PNs based on eight features. The stability and repeatability of the models were validated internally and externally. At the same time, we compared the predictive performance of our model with the Mayo model, PKUPH model and Brock model.

Age, maximum nodule diameter, nodule composition, CEA, CYFRA21‐1, P‐LCR, MCHC and MONO% were selected as the predictive features in this study. Among these factors, age, maximum nodule diameter and nodule type have been confirmed as independent risk factors for predicting PNs in multiple studies.^9^, ^10^, ^11^, ^16^, ^17^, ^18^, ^19^, ^20^, ^21^, ^22^ The risk of cancer is known to increase with age. In our study, malignant nodules were significantly larger than other nodules, which was consistent with previous studies.^23^ PNs can be classified into solid, part‐solid and pure ground‐glass types according to nodule type. Studies have reported that the malignancy rate is significantly higher for part‐solid nodules (63%) than for either solid nodules (7%) or pure ground‐glass nodules (18%); thus, nodule type may be an effective predictor for PNs.^24^ CEA and CYFRA21‐1 are widely used in the early diagnosis and prognosis prediction of lung cancer.^25^, ^26^, ^27^, ^28^ As previously reported, the serum CEA and CYFRA21‐1 levels were significantly higher in malignant PNs than in benign PNs.^29^, ^30^, ^31^ Notably, MCHC, p‐LCR and MONO% were novel predictors identified in our study, which were rarely reported in other models. This may be because previous research did not take these features into account. Therefore, more studies are needed to confirm whether these features are truly useful in predicting PNs.

The Mayo model,^9^ Brock model^10^ and PKUPH model^11^ established by the traditional LR algorithm have been widely recognized and validated. These models achieved a diagnostic accuracy of more than 80%, but the predictive performance for our patients was poor. The AUCs of the Mayo Model and Brock model were only 0.68 and 0.57, respectively, which might be because the two models were established based on the population with a high proportion of benign PNs, while the proportion of malignant PNs in this study was relatively high. In addition, these two models were developed based on the western population and might poorly fit the eastern population. The PKUPH model was developed based on a Chinese population, but its AUC was only 0.64 for our patients. The RF model had the best predictive performance in our study, with weighted average AUC values of 0.81 for the internal test set and 0.71 for the external validation set. RF is an ensemble learning method whose base classifier is DT. It adopts bootstrap resampling, which can effectively avoid the overfitting phenomenon, and its majority voting method can effectively improve the accuracy of classification. Compared with traditional LR algorithms, RF shows better accuracy in dealing with large‐scale and high‐dimensional data analysis tasks.

Compared with previous studies, this study had the following advantages. (1) The published models were all based on the dichotomous task to distinguish between BLs and MLs. According to the 5th edition of the World health organization (WHO) classification of thoracic tumours, lung tumours were classified as BLs, PLs and MLs, and atypical adenomatous hyperplasia (AAH) and adenocarcinoma in situ (AIS) were no longer classified as malignant tumours.^32^ Based on this, we established multiclassification prediction models, which might improve management outcomes and realize precision medicine. (2) In addition to the traditional LR model, we established three other ML models and found that the RF model performed best, which might provide a new tool for improving the predictive performance of PNs. (3) In total, 71 candidate variables were analysed in our study, which was far more than that of other studies. This helped to discover more potential risk factors related to the diagnosis of PNs. (4) Our model was developed based on multicentre data and validated internally and externally, indicating that the model had good stability and repeatability.

There were also several limitations in this study. (1) This was a retrospective analysis, and selection bias might have been present. (2) Some of the patients had incomplete data, which may have affected the results of our models. (3) The number of PLs in the external validation set was relatively small (five cases). (4) The study focused solely on samples that had pathological results. This limited the inclusiveness of the study and its applicability to the wider population. To enhance the effectiveness and generalizability of the model, future research should involve more prospective, large‐sample, multicenter and diverse data to fully validate the model's efficacy. (5) We did not create a prediction model using ensemble learning techniques, which could have possibly limited the extent to which we could enhance the performance of the model. Future work will explore the utilization of ensemble models like Bagging and Boosting to potentially attain superior predictive accuracy.

## 5 CONCLUSIONS

In conclusion, we established four multiclassification ML models based on eight predictive features to predict the probability of malignancy of PNs. The RF model showed the best predictive performance, which is expected to replace traditional LR models and provide an noninvasive new tool for the early diagnosis of PNs.

## AUTHOR CONTRIBUTIONS

*Conception and design*: Q. Liu, X. Lv and Y. Zeng. *Administrative support*: All authors. *Provision of study materials or patients*: Q. Liu, X. Lv and D. Zhou. *Collection and assembly of data*: Q. Liu and X. Lv. *Data analysis and interpretation*: Q. Liu and X Lv. *Manuscript writing*: All authors. *Final approval of manuscript*: All authors.

## CONFLICT OF INTEREST STATEMENT

All authors have completed the ICMJE uniform disclosure form. The authors have no conflicts of interest to declare.

## ETHICAL STATEMENT

The authors are accountable for all aspects of the work in ensuring that questions related to the accuracy or integrity of any part of the work are appropriately investigated and resolved. This study was conducted in accordance with the Declaration of Helsinki (as revised in 2013). The study was approved by the Ethics Committee of the Third Affiliated Hospital of Chongqing Medical University (2022‐41), and individual consent for this retrospective analysis was waived.

## Supporting information


**Table S1.** A brief description of the three published models.
**Table S2.** Pathological diagnoses of the total cohort.
**Table S3.** The features of patients in the internal test set and external validation set.
**Table S4.** Statistics for machine learning models for the external validation set.

## Data Availability

The data that support the findings of this study are available on request from the corresponding author. The data are not publicly available due to privacy or ethical restrictions.
