# Supplementary material for: Establishment and validation of multiclassification prediction models for pulmonary nodules based on machine learning
Source: Clin Respir J. 2024 May 12;18(5):e13769. doi: 10.1111/crj.13769 (PMC11089274; doi:10.1111/crj.13769)
Supplement: Supplementary file 1 — Table S1. A brief description of the three published models. Table S2. Pathological diagnoses of the total cohort. Table S3. The features of patients in the internal test set and external validation set. Table S4. Statistics for machine learning models for the external validation set. [file CRJ-18-e13769-s001.docx]

Supplementary table 1 A brief description of the three published models

| No. | Model | Formula |
| --- | --- | --- |
| 1 | Mayo model | malignant probability = e^x^/(1 + e^x^), X = −6.8272 + (0.0391 × age) + (0.7917 × smoke) + (1.3388 × cancer) + (0.1274 × diameter) + (1.0407 × spiculation) + (0.7838 × upper lobe) |
| 2 | PKUPH model | malignant probability = e^x^/(1 + e^x^), X = −4.496 + (0.07 × age) + (0.676 × diameter) + (0.736 × spiculation) + (1.267 × family history of cancer) - (1.615 × calcification) - (1.408 × clear boundary) |
| 3 | Brock model | malignant probability = e^x^/(1 + e^x^), X = −6.6144 + (0.6467 × gender) - (5.5537 × diameter) + (0.9309 × spiculation) + (0.6009 × upper lobe) |

Where e is the natural logarithm, and predictive factors, including age (age indicates the patient's age in years), smoke (smoke indicates smoking history; 1 = yes, 0 = no), cancer (cancer indicates history of an extrathoracic cancer 5 or more years; 1 = yes, 0 = no), diameter (diameter indicates the largest nodule measurement in mm), spiculation (1 = yes, 0 = no), upper lobe (upper lobe indicates location of the nodule within the upper lobe of lung; 1 = yes, 0 = no), family history of cancer (1 = yes, 0 = no), calcification (1 = yes, 0 = no), clear boundary (1 = yes, 0 = no), gender (1 = female, 0 = male).

Supplementary table 2 Pathological diagnoses of the total cohort

| Groups | Pathological diagnoses | Number | Ratio (%) |
| --- | --- | --- | --- |
| BL group (n=250) |  |  |  |
|  | Hamartoma | 22 | 2.41 |
|  | Arteriovenous malformation | 1 | 0.11 |
|  | Tuberculoma | 60 | 6.56 |
|  | Intrapulmonary lymph nodes | 2 | 0.22 |
|  | Lung abscess | 1 | 0.11 |
|  | Pulmonary alveolar proteinosis | 1 | 0.11 |
|  | Fibrosis | 1 | 0.11 |
|  | Organizing pneumonia | 2 | 0.22 |
|  | Nodular lymphoid hyperplasia | 1 | 0.11 |
|  | Inflammatory pseudotumor | 101 | 11.05 |
|  | Bronchiolar adenoma | 2 | 0.22 |
|  | Hemangioma | 1 | 0.11 |
|  | Cryptococcus infection | 9 | 0.98 |
|  | Sclerosing pneumocytoma | 6 | 0.66 |
|  | Others | 40 | 4.38 |
| PL group (n=103) |  |  |  |
|  | Adenocarcinoma in situ | 77 | 8.42 |
|  | Atypical adenomatous hyperplasia | 26 | 2.84 |
| ML group (n=561) |  |  |  |
|  | Adenocarcinoma | 465 | 50.88 |
|  | Squamous cell carcinoma | 69 | 7.55 |
|  | Adenosquamous carcinoma | 1 | 0.11 |
|  | Small cell lung cancer | 8 | 0.88 |
|  | Atypical carcinoid tumor | 1 | 0.11 |
|  | Bronchioloalveolar carcinoma | 5 | 0.55 |
|  | Mucoepidermoid carcinoma | 3 | 0.33 |
|  | Others | 9 | 0.98 |

ML, malignant lesion; PL, precursor lesion; BL, benign lesion.

Supplementary table 3 The features of patients in the internal test set and external validation set

| **Features** | **Internal test set (n=127)** | | | **P value** | **External validation set (n=282)** | | | **P value** |
| --- | --- | --- | --- | --- | --- | --- | --- | --- |
|  | **ML group (n=72)** | **PL group (n=24)** | **BL group (n=31)** |  | **ML group (n=173)** | **PL group (n=5)** | **BL group (n=104)** |  |
| **Clinical features** |  |  |  |  |  |  |  |  |
| Gender |  |  |  | 0.62^c^ |  |  |  | 0.17^d^ |
| Female | 37 (51.4) | 15 (62.5) | 16 (51.6) |  | 77 (44.5) | 4 (80.0) | 40 (38.5) |  |
| Male | 35 (48.6) | 9 (37.5) | 15 (48.4) |  | 96 (55.5) | 1 (20.0) | 64 (61.5) |  |
| Age (years) | 59.51 ± 9.10 | 52 ± 8.40 | 56.84 ± 11.20 | 0.004^a^ | 62.32 ± 9.64 | 54.20 ± 5.67 | 57.62 ± 11.99 | 0.001^b^ |
| Smoking history |  |  |  | 0.18^c^ |  |  |  | 0.26^d^ |
| No | 42 (58.3) | 19 (79.2) | 20 (64.5) |  | 106 (61.3) | 5 (100.0) | 64 (61.5) |  |
| Yes | 30 (41.7) | 5 (20.8) | 11 (35.5) |  | 67 (38.7) | 0 (0.0) | 40 (38.5) |  |
| Number of years of smoking (years) | 0.00 (0.00 - 31.25) | 0 .00(0.00 - 0.00) | 0.00 (0.00 - 20.00) | 0.06^b^ | 0.00 (0.00 - 30.00) | 0.00 (0.00 - 0.00) | 0.00 (0.00 - 20.00) | 0.19^b^ |
| Number of cigarettes per day smoked (cigarettes) | 0.00 (0.00 - 20.00) | 0.00 (0.00 - 0.00) | 0 .00(0.00 - 9.00) | 0.19^b^ | 0 .00(0.00 - 20.00) | 0.00 (0.00 - 0.00) | 0.00 (0.00 - 20.00) | 0.22^b^ |
| Family history of cancer |  |  |  | 0.37^d^ |  |  |  | 0.36^d^ |
| No | 62 (86.1) | 23 (95.8) | 29 (93.5) |  | 162 (93.6) | 4 (80.0) | 96 (92.3) |  |
| Yes | 10 (13.9) | 1 (4.2) | 2 (6.5) |  | 11 (6.4) | 1 (20.0) | 8 (7.7) |  |
| History of hypertension |  |  |  | 0.91^c^ |  |  |  | 0.53^d^ |
| No | 57 (79.2) | 20 (83.3) | 25 (80.6) |  | 136 (78.6) | 3 (60.0) | 81 (77.9) |  |
| Yes | 15 (20.8) | 4 (16.7) | 6 (19.4) |  | 37 (21.4) | 2 (40.0) | 23 (22.1) |  |
| History of diabetes |  |  |  | 0.83^d^ |  |  |  | 0.004^d^ |
| No | 65 (90.3) | 23 (95.8) | 28 (90.3) |  | 164 (94.8) | 4 (80.0) | 87 (83.7) |  |
| Yes | 7 (9.7) | 1 (4.2) | 3 (9.7) |  | 9 (5.2) | 1 (20.0) | 17 (16.3) |  |
| History of coronary heart disease |  |  |  | 0.87^d^ |  |  |  | 0.12^d^ |
| No | 67 (93.1) | 23 (95.8) | 30 (96.8) |  | 163 (94.2) | 5 (100.0) | 103 (99.0) |  |
| Yes | 5 (6.9) | 1 (4.2) | 1 (3.2) |  | 10 (5.8) | 0 (0.0) | 1 (1.0) |  |
| History of viral hepatitis |  |  |  | 0.83^d^ |  |  |  | 0.66^d^ |
| No | 68 (94.4) | 24 (100.0) | 30 (96.8) |  | 166 (96.0) | 5 (100.0) | 98 (94.2) |  |
| Yes | 4 (5.6) | 0 (0.0) | 1 (3.2) |  | 7 (4.0) | 0 (0.0) | 6 (5.8) |  |
| History of hyperlipidemia |  |  |  | 0.90^d^ |  |  |  | 0.006^d^ |
| No | 66 (91.7) | 23 (95.8) | 29 (93.5) |  | 170 (98.3) | 3 (60.0) | 102 (98.1) |  |
| Yes | 6 (8.3) | 1 (4.2) | 2 (6.5) |  | 3 (1.7) | 2 (40.0) | 2 (1.9) |  |
| History of pulmonary tuberculosis |  |  |  | 0.19^d^ |  |  |  | >0.99^d^ |
| No | 72 (100.0) | 23 (95.8) | 30 (96.8) |  | 165 (95.4) | 5 (100.0) | 100 (96.2) |  |
| Yes | 0 (0.0) | 1 (4.2) | 1 (3.2) |  | 8 (4.6) | 0 (0.0) | 4 (3.8) |  |
| History of chronic obstructive pulmonary disease |  |  |  | 0.24^d^ |  |  |  | 0.40^d^ |
| No | 71 (98.6) | 24 (100.0) | 29 (93.5) |  | 162 (93.6) | 4 (80.0) | 97 (93.3) |  |
| Yes | 1 (1.4) | 0 (0.0) | 2 (6.5) |  | 11 (6.4) | 1 (20.0) | 7 (6.7) |  |
| History of cancer |  |  |  | / |  |  |  | >0.99^d^ |
| No | 72 (100.0) | 24 (100.0) | 31 (100.0) |  | 168 (97.1) | 5 (100.0) | 101 (97.1) |  |
| Yes | 0 (0.0) | 0 (0.0) | 0 (0.0) |  | 5 (2.9) | 0 (0.0) | 3 (2.9) |  |
| Systolic blood pressure (mmHg) | 129.40 ± 20.70 | 126.38 ± 19.40 | 122.70 ± 19.25 | 0.30^a^ | 130.44 ± 17.15 | 134.80 ± 7.05 | 127.68 ± 17.75 | 0.35^a^ |
| Diastolic blood pressure (mmHg) | 76.56 ± 11.82 | 73.33 ± 11.96 | 76.47 ± 13.81 | 0.52^a^ | 78.68 ± 11.24 | 88.60 ± 6.15 | 77.73 ± 9.95 | 0.08^a^ |
| Pulse (beats/minute) | 76.65 ± 12.88 | 75.00 ± 8.19 | 75.42 ± 8.66 | 0.77^a^ | 80.23 ± 11.59 | 80.80 ± 5.12 | 82.45 ± 11.86 | 0.31^a^ |
| Temperature (℃) | 36.50 (36.40 - 36.60) | 36.50 (36.40 - 36.60) | 36.6 (36.40 - 36.70) | 0.55^b^ | 36.50 (36.40 - 36.70) | 36.60 (36.50 - 36.60) | 36.50 (36.40 - 36.62) | 0.71^b^ |
| Respiratory rate (breaths/minute) | 20 (19.0 - 20.0) | 20 (18.8 - 20.0) | 20 (18.0 - 20.0) | 0.52^b^ | 20 (19.0 - 20.0) | 19 (19.0 - 20.0) | 20 (20.0 - 20.0) | 0.39^b^ |
| **Radiologic features** |  |  |  |  |  |  |  |  |
| Nodule location |  |  |  | 0.15^d^ |  |  |  | 0.50^d^ |
| Upper right | 29 (40.3) | 7 (29.2) | 8 (25.8) |  | 50 (28.9) | 4 (80.0) | 23 (22.1) |  |
| Lower right | 10 (13.9) | 2 (8.3) | 7 (22.6) |  | 39 (22.5) | 0 (0.0) | 24 (23.1) |  |
| Middle right | 4 (5.6) | 6 (25.0) | 3 (9.7) |  | 8 (4.6) | 0 (0.0) | 6 (5.8) |  |
| Upper left | 13 (18.1) | 6 (25.0) | 4 (12.9) |  | 46 (26.6) | 1 (20.0) | 29 (27.9) |  |
| Lower left | 16 (22.2) | 3 (12.5) | 9 (29.0) |  | 30 (17.3) | 0 (0.0) | 22 (21.2) |  |
| Nodule type |  |  |  | <0.001^c^ |  |  |  | <0.001^d^ |
| Solid | 36 (50.0) | 1 (4.2) | 25 (80.6) |  | 153 (88.4) | 0 (0.0) | 94 (90.4) |  |
| Part-solid | 21 (29.2) | 8 (33.3) | 3 (9.7) |  | 12 (6.9) | 0 (0.0) | 5 (4.8) |  |
| Pure ground-glass | 15 (20.8) | 15 (62.5) | 3 (9.7) |  | 8 (4.6) | 5 (100.0) | 5 (4.8) |  |
| Maximal diameter of nodule, mm | 17.41 ± 6.06 | 10.01 ± 3.27 | 12.69 ± 6.18 | <0.001^b^ | 20.59 ± 5.26 | 9.80 ± 2.59 | 16.94 ± 6.39 | <0.001^b^ |
| Clear boundary |  |  |  | 0.23^d^ |  |  |  | 0.04^d^ |
| No | 64 (88.9) | 22 (91.7) | 24 (77.4) |  | 137 (79.2) | 4 (80.0) | 68 (65.4) |  |
| Yes | 8 (11.1) | 2 (8.3) | 7 (22.6) |  | 36 (20.8) | 1 (20.0) | 36 (34.6) |  |
| Regular edges |  |  |  | 0.54^d^ |  |  |  | 0.001^d^ |
| No | 67 (93.1) | 23 (95.8) | 27 (87.1) |  | 165 (95.4) | 4 (80.0) | 85 (81.7) |  |
| Yes | 5 (6.9) | 1 (4.2) | 4 (12.9) |  | 8 (4.6) | 1 (20.0) | 19 (18.3) |  |
| Shaggy boundary |  |  |  | 0.58^d^ |  |  |  | 0.24^d^ |
| No | 69 (95.8) | 24 (100.0) | 31 (100.0) |  | 144 (83.2) | 5 (100.0) | 94 (90.4) |  |
| Yes | 3 (4.2) | 0 (0.0) | 0 (0.0) |  | 29 (16.8) | 0 (0.0) | 10 (9.6) |  |
| Lobulation |  |  |  | 0.03^c^ |  |  |  | <0.001^d^ |
| No | 54 (75.0) | 23 (95.8) | 28 (90.3) |  | 100 (57.8) | 3 (60.0) | 88 (84.6) |  |
| Yes | 18 (25.0) | 1 (4.2) | 3 (9.7) |  | 73 (42.2) | 2 (40.0) | 16 (15.4) |  |
| Spiculation |  |  |  | 0.11^c^ |  |  |  | <0.001^d^ |
| No | 51 (70.8) | 22 (91.7) | 22 (71.0) |  | 96 (55.5) | 5 (100.0) | 84 (80.8) |  |
| Yes | 21 (29.2) | 2 (8.3) | 9 (29.0) |  | 77 (44.5) | 0 (0.0) | 20 (19.2) |  |
| Pleural indentation |  |  |  | 0.04^c^ |  |  |  | 0.005^d^ |
| No | 56 (77.8) | 24 (100.0) | 26 (83.9) |  | 113 (65.3) | 5 (100.0) | 85 (81.7) |  |
| Yes | 16 (22.2) | 0 (0.0) | 5 (16.1) |  | 60 (34.7) | 0 (0.0) | 19 (18.3) |  |
| Adjacent pleural thickening |  |  |  | 0.68^d^ |  |  |  | 0.33^d^ |
| No | 71 (98.6) | 24 (100.0) | 30 (96.8) |  | 163 (94.2) | 5 (100.0) | 93 (89.4) |  |
| Yes | 1 (1.4) | 0 (0.0) | 1 (3.2) |  | 10 (5.8) | 0 (0.0) | 11 (10.6) |  |
| Vacuole sign |  |  |  | 0.33^d^ |  |  |  | 0.51^d^ |
| No | 60 (83.3) | 22 (91.7) | 29 (93.5) |  | 157 (90.8) | 4 (80.0) | 95 (91.3) |  |
| Yes | 12 (16.7) | 2 (8.3) | 2 (6.5) |  | 16 (9.2) | 1 (20.0) | 9 (8.7) |  |
| Cavity |  |  |  | >0.99**^d^** |  |  |  | 0.09^d^ |
| No | 71 (98.6) | 24 (100.0) | 31 (100.0) |  | 168 (97.1) | 5 (100.0) | 95 (91.3) |  |
| Yes | 1 (1.4) | 0 (0.0) | 0 (0.0) |  | 5 (2.9) | 0 (0.0) | 9 (8.7) |  |
| Calcification |  |  |  | 0.24^d^ |  |  |  | 0.003^d^ |
| No | 71 (98.6) | 24 (100.0) | 29 (93.5) |  | 170 (98.3) | 5 (100.0) | 92 (88.5) |  |
| Yes | 1 (1.4) | 0 (0.0) | 2 (6.5) |  | 3 (1.7) | 0 (0.0) | 12 (11.5) |  |
| Enlargement of hilar or mediastinal lymph nodes |  |  |  | 0.22^d^ |  |  |  | 0.11^d^ |
| No | 63 (87.5) | 24 (100.0) | 28 (90.3) |  | 147 (85.0) | 5 (100.0) | 97 (93.3) |  |
| Yes | 9 (12.5) | 0 (0.0) | 3 (9.7) |  | 26 (15.0) | 0 (0.0) | 7 (6.7) |  |
| Number of nodule |  |  |  | 0.25^c^ |  |  |  | 0.31^d^ |
| Solitary | 25 (34.7) | 5 (20.8) | 13 (41.9) |  | 90 (52.0) | 2 (40.0) | 45 (43.3) |  |
| Multiple | 47 (65.3) | 19 (79.2) | 18 (58.1) |  | 83 (48.0) | 3 (60.0) | 59 (56.7) |  |
| **Laboratory test** |  |  |  |  |  |  |  |  |
| CEA (ng/ml) | 2.61 (1.61 - 3.76) | 1.82 (1.29 - 2.75) | 2.24 (1.53 - 3.36) | 0.04^b^ | 3.12 (1.86 - 3.76) | 2.16 (1.22 - 2.27) | 1.98 (1.19 - 3.17) | <0.001^b^ |
| Pro-GRP (pg/ml) | 51.38 (39.33 - 57.22) | 48.37 (41.40 - 56.73) | 57.22 (46.77 - 57.29) | 0.31^b^ | 57.22 (36.23 - 57.22) | 57.22 (57.22 - 57.22) | 57.22 (30.66 - 57.22) | 0.58^b^ |
| NSE (ng/ml) | 15.93 (12.14 - 18.11) | 14.97 (11.00 - 17.51) | 17.22 (11.61 - 17.24) | 0.82^b^ | 11.72 (8.72 - 17.24) | 9.66 (9.03 - 10.66) | 10.21 (7.33 - 14.27) | 0.06^b^ |
| CYFRA21-1 (ng/ml) | 2.29 (1.59 - 2.74) | 1.85 (1.40 - 2.47) | 2.25 (1.84 - 2.78) | 0.26^b^ | 2.31 (1.67 - 3.14) | 2.33 (2.19 - 3.07) | 2.22 (1.29 - 2.46) | 0.07^b^ |
| P-LCR (%) | 31.30 ± 9.12 | 34.02 ± 11.75 | 29.29 ± 9.26 | 0.20^a^ | 31.08 (31.08 - 35) | 31.08 (31.08 - 31.08) | 31.08 (31.01 - 32.55) | 0.77^b^ |
| P-LCC (10^9/L) | 59.46 ± 13.30 | 63.58 ± 16.82 | 60.42 ± 18.38 | 0.52^a^ | 59.64 (59.64 - 59.64) | 59.64 (59.64 - 59.64) | 59.64 (59.64 - 59.64) | 0.25^b^ |
| MONO% (%) | 5.71 ± 1.63 | 6.06 ± 1.33 | 6.35 ± 1.60 | 0.15^a^ | 5.80 (4.80 - 7.40) | 6.70 (6.20 - 8.10) | 6 (4.77 - 7.03) | 0.63^b^ |
| RDW-CV (%) | 13.00 (12.60 - 13.62) | 12.75 (12.47 - 13.20) | 12.80 (12.60 - 13.35) | 0.23^b^ | 13.20 (12.60 - 13.80) | 14.10 (13.40 - 14.30) | 12.90 (12.50 - 13.62) | 0.03^b^ |
| RDW-SD (fL) | 44.95 (42.20 - 47.12) | 44.40 (43.77 - 46.65) | 45.90 (44.15 - 46.75) | 0.54^b^ | 44.29 (42.70 - 44.60) | 44.29 (44.29 - 44.29) | 44.29 (42.60 - 45.12) | 0.99^b^ |
| HCT (%) | 40.86 ± 4.53 | 42.49 ± 3.90 | 42.01 ± 3.86 | 0.19^a^ | 40.97 ± 4.20 | 42.08 ± 5.67 | 41.85 ± 5.16 | 0.28^a^ |
| LYM% (%) | 25.77 ± 7.96 | 29.77 ± 7.29 | 30.55 ± 7.42 | 0.007^a^ | 24.14 ± 7.77 | 24.32 ± 1.72 | 24.34 ± 8.29 | 0.98^b^ |
| MCV (fL) | 92.80 (90.47 - 95.60) | 93.45 (91.62 - 96.58) | 95.40 (92.00 - 98.00) | 0.07^b^ | 92.37 (89.60 - 96.00) | 95.00 (92.10 - 99.00) | 92.00 (89.45 - 95.00) | 0.69^b^ |
| MCH (pg) | 30.75 (29.60 - 31.50) | 30.60 (29.45 - 31.55) | 30.60 (30.20 - 32.20) | 0.52^b^ | 30.40 (29.40 - 31.30) | 30.20 (30.10 - 30.60) | 30.40 (29.30 - 31.50) | 0.79^b^ |
| MCHC (g/L) | 328.14 ± 11.25 | 325.96 ± 9.33 | 327.81 ± 8.86 | 0.67^a^ | 327.00 (320.00 - 335.00) | 310.00 (304.00 - 322.00) | 329.00 (323.75 - 335.25) | 0.04^b^ |
| MPV (fL) | 10.67 ± 1.31 | 11.19 ± 1.70 | 10.44 ± 1.34 | 0.14^a^ | 11.21 ± 1.31 | 11.60 ± 1.27 | 11.10 ± 1.29 | 0.61^a^ |
| BAS% (%) | 0.40 (0.30 - 0.60) | 0.40 (0.30 - 0.60) | 0.50 (0.30 - 0.60) | 0.58^b^ | 0.40 (0.20 - 0.50) | 0.40 (0.30 - 0.50) | 0.30 (0.20 - 0.50) | 0.68^b^ |
| EOS% (%) | 1.85 (0.90 - 3.42) | 1.90 (1.00 - 2.97) | 2.20 (1.40 - 4.25) | 0.44^b^ | 1.90 (1.00 - 3.00) | 3 .00(1.50 - 3.10) | 1.50 (0.80 - 2.50) | 0.35^b^ |
| PDW (%) | 16.26 ± 0.39 | 16.20 ± 0.29 | 16.20 ± 0.33 | 0.69^a^ | 16.00 (14.00 - 16.40) | 16.30 (15.00 - 16.50) | 16.20 (13.97 - 16.52) | 0.51^b^ |
| PCT (%) | 0.21 ± 0.05 | 0.21 ± 0.04 | 0.22 ± 0.06 | 0.60^a^ | 0.21 (0.19 - 0.26) | 0.19 (0.17 - 0.22) | 0.23 (0.19 - 0.27) | 0.25^b^ |
| NEU% (%) | 65.16 ± 9.26 | 61.55 ± 8.07 | 59.41 ± 8.70 | 0.009^a^ | 66.52 ± 9.18 | 64.48 ± 5.87 | 66.85 ± 9.49 | 0.84^a^ |
| WBC (10^9/L) | 5.92 ± 1.80 | 5.45 ± 1.29 | 5.57 ± 1.58 | 0.42^b^ | 6.01 (5.04 - 7.28) | 4.30 (4.22 - 5.80) | 6.26 (5.18 - 7.76) | 0.21^b^ |
| EOS# (10^9/L) | 0.09 (0.05 - 0.19) | 0.08 (0.06 - 0.16) | 0.11 (0.08 - 0.21) | 0.39^b^ | 0.12 (0.06 - 0.17) | 0.12 (0.07 - 0.23) | 0.10 (0.05 - 0.18) | 0.64^b^ |
| BAS# (10^9/L) | 0.03 (0.01 - 0.03) | 0.02 (0.02 - 0.03) | 0.03 (0.02 - 0.04) | 0.57^b^ | 0.02 (0.01 - 0.03) | 0.02 (0.02 - 0.02) | 0.02 (0.01 - 0.03) | 0.53^b^ |
| NEU# (10^9/L) | 3.89 ± 1.55 | 3.38 ± 1.03 | 3.36 ± 1.17 | 0.10^a^ | 3.96 (3.17 - 5.05) | 2.97 (2.50 - 3.82) | 4.16 (3.12 - 5.55) | 0.28^b^ |
| LYM# (10^9/L) | 1.46 ± 0.53 | 1.60 ± 0.43 | 1.67 ± 0.60 | 0.15^a^ | 1.46 (1.13 - 1.70) | 1.15 (1.00 - 1.40) | 1.44 (1.13 - 1.84) | 0.44^b^ |
| MONO# (10^9/L) | 0.33 ± 0.11 | 0.33 ± 0.11 | 0.35 ± 0.13 | 0.69^a^ | 0.36 (0.27 - 0.46) | 0.32 (0.27 - 0.50) | 0.37 (0.28 - 0.47) | 0.88^b^ |
| PLT (10^9/L) | 202.93 ± 62.59 | 196.79 ± 49.60 | 213.68 ± 59.49 | 0.56^a^ | 204.76 ± 66.61 | 169.80 ± 35.58 | 209.83 ± 61.35 | 0.37^a^ |
| HGB (g/L) | 133.79 ± 17.22 | 138.38 ± 13.14 | 137.68 ± 12.31 | 0.32^a^ | 134.11 ± 14.41 | 131.00 ± 20.43 | 137.10 ± 16.58 | 0.24^a^ |
| RBC (10^12/L) | 4.46 ± 0.51 | 4.49 ± 0.52 | 4.46 ± 0.46 | 0.96^a^ | 4.49 ± 0.52 | 4.66 ± 0.79 | 4.58 ± 0.65 | 0.38^a^ |
| APTT (s) | 30.95 ± 3.05 | 31.28 ± 3.14 | 30.17 ± 2.45 | 0.33^a^ | 31.04 ± 5.92 | 26.20 ± 2.52 | 31.00 ± 6.48 | 0.22^a^ |
| TT (s) | 14.54 ± 1.23 | 14.80 ± 1.08 | 14.61 ± 1.29 | 0.65^a^ | 17.30 (15.80- 18.60) | 14.63 (14.63 - 15.61) | 16.80 (14.76 - 17.72) | 0.04^b^ |
| PTA (%) | 103.04 ± 14.77 | 104.96 ± 12.21 | 105.42 ± 10.00 | 0.71^b^ | 108.73 ± 16.98 | 110.26 ± 6.30 | 106.04 ± 17.69 | 0.43^a^ |
| PT (s) | 10.95 (10.60 - 11.72) | 10.90 (10.57 - 11.45) | 10.90 (10.50 - 11.20) | 0.75^b^ | 11.90 (11.10 - 12.90) | 11.30 (10.90 - 11.30) | 12.15 (11.10 - 12.90) | 0.15^b^ |
| FIB (g/L) | 2.95 ± 0.68 | 2.83 ± 0.56 | 2.91 ± 0.61 | 0.74^a^ | 2.99 (2.49 - 3.57) | 3.00 (2.40 - 3.10) | 3 .00(2.46 - 3.76) | 0.85^b^ |
| D-DimerHS (ng/ml) | 91.50 (51.25 - 134) | 75 (56.75 - 96.50) | 94 (54.50 - 119.90) | 0.25^b^ | 119.90 (119.90 - 119.90) | 119.90 (119.90 - 119.90) | 119.90 (119.90 - 119.90) | 0.43^b^ |
| INR | 0.96 (0.93 - 1.03) | 0.96 (0.93 - 1.00) | 0.96 (0.92 - 0.98) | 0.75^b^ | 0.97 (0.93 - 1.02) | 0.94 (0.94 - 0.97) | 0.98 (0.94 - 1.03) | 0.33^b^ |
| ALB (g/L) | 43.77 ± 4.17 | 43.73 ± 4.10 | 42.99 ± 3.23 | 0.64^a^ | 43.79 (40.80 - 43.79) | 43.79 (43.79 - 43.79) | 43.79 (41.45 - 43.79) | 0.22^b^ |

Data are expressed as mean ± standard deviation or medians and interquartile range or counts with percentages.

^a^, calculated using analysis of variance (ANOVA) test; ^b^, calculated using Kruskal-Wallis test; ^c^, calculated using Chi-square test; ^d^, calculated using Fisher's exact test.

ML, malignant lesion; PL, precursor lesion; BL, benign lesion; CEA, carcinoembryonic antigen; ProGRP, pro-gastrin-releasing peptide; NSE, neuron-specific enolase; CYFRA21-1, cytokeratin 19 fragment; P-LCR, platelet large cell ratio; P-LCC, platelet large cell count; MONO%, percentage of monocytes; RDW-CV, red blood cell distribution width coefficient variation; RDW-SD, red blood cell distribution width standard deviation; HCT, hematocrit; LYM%, percentage of lymphocytes; MCV, mean corpuscular volume; MCH, mean corpuscular hemoglobin; MCHC, mean corpuscular hemoglobin concentration; MPV, mean platelet volume; BAS%, percentage of basophils; EOS%, percentage of eosinophils; PDW, platelet distribution width; PCT, plateletcrit; NEU%, percentage of neutrophil; WBC, white blood cell; EOS, eosinophil count; BAS, basophil count; NEU, neutrophil count; LYM, lymphocyte count; MONO, monocyte count; PLT, platelets; HGB, hemoglobin; RBC, red blood cell; APTT, activated partial thromboplastin time; TT, thrombin time; PTA, prothrombin activity; PT, prothrombin time; FIB fibrinogen; D-DimerHS, Hypersensitive D-Dimer; INR, international normalized ratio; ALB, albumin.

Supplementary table 4 Statistics for machine learning models for the external validation set

| Model | Type | Accuracy | Precision | Recall | F1-score | AUC(95% CI) |
| --- | --- | --- | --- | --- | --- | --- |
| LR |  | 0.66 |  |  |  |  |
|  | MLs |  | 0.66 | 0.94 | 0.78 | 0.72(0.66-0.78) |
|  | PLs |  | 0.00 | 0.00 | 0.00 | 0.98(0.90-1.07) |
|  | BLs |  | 0.73 | 0.23 | 0.35 | 0.67(0.61-0.74) |
|  | Weighted average |  | 0.67 | 0.66 | 0.61 | 0.70(0.67-0.73) |
| DT |  | 0.61 |  |  |  |  |
|  | MLs |  | 0.69 | 0.71 | 0.70 | 0.63(0.54-0.67) |
|  | PLs |  | 0.25 | 0.60 | 0.35 | 0.93(0.65-1.07) |
|  | BLs |  | 0.50 | 0.45 | 0.47 | 0.59(0.52-0.66) |
|  | Weighted average |  | 0.61 | 0.61 | 0.61 | 0.62(0.55-0.67) |
| SVM |  | 0.62 |  |  |  |  |
|  | MLs |  | 0.73 | 0.62 | 0.67 | 0.71(0.62-0.74) |
|  | PLs |  | 0.40 | 0.80 | 0.53 | 0.99(0.93-1.05) |
|  | BLs |  | 0.50 | 0.60 | 0.54 | 0.65(0.60-0.73) |
|  | Weighted average |  | 0.64 | 0.62 | 0.62 | 0.69(0.64-0.70) |

AUC, area under the curve; 95% CI, 95% confidence interval; LR, logical regression; DT, decision tree; SVM, support vector machine; MLs, malignant lesions; PLs, precursor lesions; BLs, benign lesions.
